# Protective effects of Araloside C against myocardial ischaemia/reperfusion injury: potential involvement of heat shock protein 90

**DOI:** 10.1111/jcmm.13107

**Published:** 2017-02-22

**Authors:** Min Wang, Yu Tian, Yu‐yang Du, Gui‐bo Sun, Xu‐dong Xu, Hai Jiang, Hui‐bo Xu, Xiang‐bao Meng, Jing‐yi Zhang, Shi‐lan Ding, Miao‐di Zhang, Ming‐hua Yang, Xiao‐bo Sun

**Affiliations:** ^1^ Beijing Key Laboratory of Innovative Drug Discovery of Traditional Chinese Medicine (Natural Medicine) and Translational Medicine Institute of Medicinal Plant Development Chinese Academy of Medical Sciences & Peking Union Medical College Beijing China; ^2^ Key Laboratory of Chinese Materia Medica (Ministry of Education) Heilongjiang University of Chinese Medicine Harbin Heilongjang China; ^3^ Academy of Chinese Medical Sciences of Jilin Province Changchun Jilin China; ^4^ Harbin University of Commerce Harbin Heilongjiang China

**Keywords:** Araloside C, ischaemia/reperfusion, heat shock protein 90, biomolecular interactions

## Abstract

The present study was designed to investigate whether Araloside C, one of the major triterpenoid compounds isolated from *Aralia elata* known to be cardioprotective, can improve heart function following ischaemia/reperfusion (I/R) injury and elucidate its underlying mechanisms. We observed that Araloside C concentration‐dependently improved cardiac function and depressed oxidative stress induced by I/R. Similar protection was confirmed in isolated cardiomyocytes characterized by maintaining Ca^2+^ transients and cell shortening against I/R. Moreover, the potential targets of Araloside C were predicted using the DDI‐CPI server and Discovery Studio software. Molecular docking analysis revealed that Araloside C could be stably docked into the ATP/ADP‐binding domain of the heat shock protein 90 (Hsp90) protein via the formation of hydrogen bonds. The binding affinity of Hsp90 to Araloside C was detected using nanopore optical interferometry and yielded KD values of 29 μM. Araloside C also up‐regulated the expression levels of Hsp90 and improved cell viability in hypoxia/reoxygenation‐treated H9c2 cardiomyocytes, whereas the addition of 17‐AAG, a pharmacologic inhibitor of Hsp90, attenuated Araloside C‐induced cardioprotective effect. These findings reveal that Araloside C can efficiently attenuate myocardial I/R injury by reducing I/R‐induced oxidative stress and [Ca^2+^]_i_ overload, which was possibly related to its binding to the Hsp90 protein.

## Introduction

Myocardial ischaemia/reperfusion injury (MIRI), which causes increased myocardial dysfunction and further cardiomyocyte death, is considered to be one of the leading causes of mortality and morbidity after cardiac operations and myocardial infarctions 1. The pathogenesis of I/R injury is complicated and multi‐factorial, which primarily includes excessive reactive oxygen species (ROS) production and intracellular calcium overload 2, 3. In the past several decades, although several pharmacological and mechanical approaches (such as ischaemic pre‐ or post‐conditioning) have been developed to limit myocardial I/R injury, this disease is still a major clinical challenge with life‐threatening outcomes 4, 5. Therefore, it is necessary to further develop novel therapeutic agents and strategies in the treatment of MIRI.

Heat shock protein (Hsp) 90, as a highly abundant and ubiquitous molecular chaperone, has been found to interact with a large number of client proteins, including members of the Src kinase family of non‐receptor tyrosine kinases, Raf and other serine/threonine kinases, transcription factors such as steroid hormone receptors and p53, and eNOS 6. In recent years, studies have shown that HSP90 may confer a cardioprotective effect against ischaemia/reperfusion injury 7, 8. Targeted overexpression of Hsp90 in myocardium reduces infarct size and enhances the association between Hsp90, eNOS and Akt 9. Besides, compounds that bind to HSP90 or disrupt Hsp90 chaperone complex, such as radicicol, could induce HSP90 expression and result in the protection against simulated ischaemia/reperfusion injury in cardiomyocytes 10. Thus, modulation of the active HSP90 protein level may contribute to the potential therapeutic benefits for the treatment of I/R injury.

**Figure 1:**
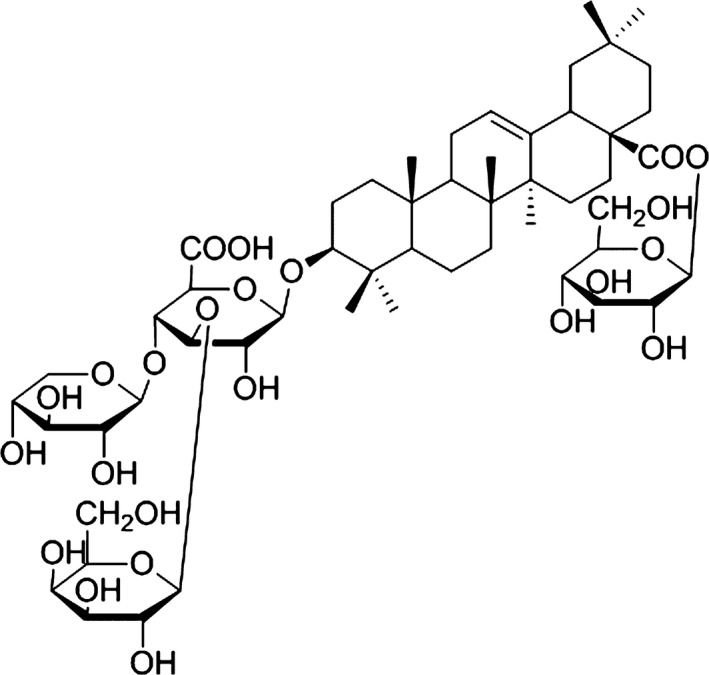
The chemical structure of Araloside C.

*Aralia elata* (Miq) Seem, a well‐known adaptogenic plant, has been traditionally used as a tonic herb to increase energy and improve the body's ability to prevent hypoxia 11, 12. The saponins of *Aralia elata* (AS) are considered the main pharmacologically active ingredient extracted from *A. elata* 13. Our previous studies confirmed that AS exhibits anti‐myocardial ischaemic and anti‐hypoxic activities 14, 15. Moreover, *A. elata* Xinmaitong capsules (Clinical Trial Approval Number 2003L01111 from China Food and Drug Administration), which we developed for the treatment of coronary heart disease, are mainly composed of AS 11. Various oleanane‐type triterpene saponins were isolated and identified from AS, which might contribute to the cardioprotective effects of AS 16, 17, 18. For example, Elatoside C was recently reported to significantly reduce cardiac injury during I/R and alleviate hypoxia/reoxygenation (H/R)‐induced cardiomyocyte apoptosis 19, 20. Thus, it is interesting to explore the active compounds from AS with cardioprotective potentials. Araloside C (Fig. 1), one of the most abundant triterpenoid compounds isolated from *A. elata*, has been previously indicated to distinctly stimulate heart activity 21, 22. However, the cardioprotective properties and underlying mechanisms of Araloside C are largely unknown.

Therefore, the present study was designed to determine whether Araloside C exerts cardioprotective effects in myocardial I/R injury and to investigate the molecular targets underlying the cardioprotective effects of Araloside C.

## Materials and methods

### Materials

Araloside C was obtained from the Institute of Medicinal Plant Development (Beijing, China); for details, see Supplementary material. Collagenase Type II and Fura‐2/AM were purchased from Life Technologies Corporation (Carlsbad, CA, USA). The kits for determining malondialdehyde (MDA) content, glutathione peroxidase (GSH‐Px) activity and superoxide dismutase (SOD) activity were obtained from Jiancheng Bioengineering Institute (Nanjing, China). Cell culture products were purchased from Gibco BRL (Grand Island, NY, USA). MTT [3‐(4,5‐dimethylthiazol‐2‐yl)‐2,5‐diphenyltetrazolium bromide] and 17‐allylamino‐17‐demethoxygeldanamycin (17‐AAG) were the products of Sigma Chemical Co. (St. Louis, MO, USA). The total ROS detection kit was obtained from Enzo Life Sciences (Farmingdale, NY, USA). Primary antibodies against Hsp90 and β‐actin were obtained from Abcam (Cambridge, UK). Horseradish peroxidase (HRP)‐conjugated secondary antibodies were secured from CW Biotech (Beijing, China). All chemical reagents were of at least analytical grade.

### Animals and ethics

Male Sprague–Dawley rats (180–250 g) were purchased from Beijing Vital River Laboratory Animal Technology Co., Ltd., Beijing, China. The animals were housed under standard laboratory conditions (25 ± 1°C, 60% humidity, with a 12‐hrs photoperiod) and provided free access to sterile food and water. All of the procedures were approved by the Laboratory Animal Ethics Committee of the Institute of Medicinal Plant Development, Peking Union Medical College, and conformed to the Guide for the Care and Use of Laboratory Animals (NIH publication No. 86‐23, revised 1996).

### Isolated rat heart Langendorff perfusion

As described in our previous study 20, male Sprague–Dawley rats were anesthetized with sodium pentobarbital (45 mg/kg i.p.) and heparinized. Next, the heart was rapidly excised and cannulated on a Langendorff perfusion system through the aorta and perfused at a constant pressure of 80 mmHg with Krebs–Henseleit (KH) buffer (37°C, pH 7.4), which was constantly gassed with 95% O_2_ and 5% CO_2_. The left ventricular pressure and heart rate (HR) were measured via a pressure transducer (AD Instruments, Sydney, NSW, Australia) that was connected to a water‐filled wrap balloon inserted into the left ventricle, achieving a left ventricular end‐diastolic pressure (LVEDP) between 2 and 8 mmHg. The isolated heart was surrounded by a homoeothermic glass cover (37°C) to maintain temperature. All of the data were recorded using PowerLab and analysed using Chart V 7.3.3 (AD Instruments).

### Experimental protocols in isolated hearts

All of the hearts were stabilized with KH buffer for a period of 30 min. before the application of the experimental protocols described in this study. To determine the concentration–response relationship of Araloside C on the recovery of post‐ischaemic myocardial function, the hearts were randomly divided into the following experimental groups (*n* = 15 hearts/group): (*i*) control, the isolated hearts were perfused for 90 min. with oxygenated KHB; (*ii*) I/R, the isolated hearts were perfused with KHB for 15 min. and then subjected to 45 min. of no‐flow global ischaemia and 30 min. of reperfusion; and (*iii*) Araloside C + I/R, the isolated hearts were perfused with KHB containing Araloside C at concentrations of 0.5, 1 and 2.5 μM for 15 min. prior to I/R.

### Determination of oxidative stress‐related indicators in isolated hearts

The left ventricle was harvested, and myocardial homogenates were prepared for the detection of MDA, GSH‐Px and SOD by the corresponding kits (Nanjing Jiancheng Bioengineering Institute, Nanjing, China) as per the manufacturer's instructions. The activities of NADPH oxidase were investigated by NADPH Oxidase Activity Detection kit (Genmed Scientifics INC., Shanghai, China). ROS levels were detected by the Rat ROS ELISA kit (RapidBio Lab, Calabasas, CA, USA).

### Isolation of adult rat ventricular myocytes

Individual rat left ventricular myocytes were isolated using an enzymatic method as previously reported 11. Only isolated rod‐shaped myocytes accounting for >85% at the beginning of each experiment were considered satisfactory.

### Simultaneous measurement of Ca^2+^ transients and sarcomere shortening

Ca^2+^ transients and sarcomere shortening were detected simultaneously using a video‐based sarcomere length and Ca^2+^ acquisition module system (IonOptix Corporation, Milton, MA, USA), as previously described 11. Cardiomyocytes were incubated with Fura‐2 AM (2 μM for 20 min. at 37°C; Invitrogen, Carlsbad, CA, USA). The loaded cells were electrically stimulated at 0.5 Hz. The ratio of fluorescence emitted at 340 and 380 nm was recorded as an indicator of [Ca^2+^]_i_. The data were recorded and analysed with IonWizard software (version 6.2.0.59, IonOptix LLC, Milton, MA, USA).

### Experimental protocols in isolated cardiomyocytes

A cellular model of simulated I/R was used as previously described 20. Briefly, control recordings were made for 15 min. in normal Tyrode's solution at 37°C, pH 7.4. Subsequently, the solution was switched to an ischaemic solution (in mM: 123 NaCl, 6 NaHCO_3_, 0.9 NaH_2_PO_4_, 8 KCl, 0.5 MgSO_4_, 20 Na lactate and 1.8 CaCl_2_; pH 6.8 and equilibrated with 90% N_2_–10% CO_2_) for 20 min. followed by a 30‐min. reperfusion with normal Tyrode's solution. The effects of Araloside C on Ca^2+^ transients and sarcomere shortening during I/R were examined by perfusing cells with Araloside C (2–8 μM) for 5 min. prior to ischaemia. Time controls were exposed to normal Tyrode's solution for 65 min. without ischaemia.

### Prediction of the targets of Araloside C by bioinformatic approach

#### Targets predicted by the CPI

The interaction information of Araloside C was predicted using the DDI‐CPI tool, a web‐based server that can predict drug–drug interactions via the chemical–protein interactome. The molecular file of Araloside C was downloaded and pre‐treated following the web instructions. Next, the file was submitted to the DDI‐CPI servers. Parameters were set to default values.

#### Targets predicted by Discovery Studio 4.5

The molecular targets of Araloside C were predicted using Discovery Studio 4.5 (BIOVIA Software Inc., San Diego, CA, USA), a software suite for performing computational analysis of data relevant to Life Sciences research. To determine the proper target of Araloside C, we employed the Ligand Profiler protocol which maps a set of pharmacophores, including Pharma DB by default. The ligand of Araloside was prepared by the Specifying Ligands parameter protocol. After inputting all parameters, the job was run and the results were monitored from the Jobs Explorer.

### Molecular docking

To explore the potential interacting mode of Araloside C with the Hsp90 protein (PDB code: 1AMW), a molecular modelling study was performed using the docking program named Lib Dock, a software package in Discovery Studio 4.5. To eliminate any bond length and bond angle biases, the ligand (Araloside C) was subjected to a full minimization prior to docking. The binding affinities (LibDockScore) in Discovery Studio 4.5 were used to evaluate the interactions between Hsp90 and Araloside C.

### Nanopore optical interferometry

Label‐free molecule/protein interaction detection and kinetic constant measurement were studied using the SKi Pro System (Silicon Kinetics, San Diego, CA, USA) 23. Nanoporous carboxy chips were activated using sulpho‐NHS/EDAC chemistry in a buffer consisting of 0.1 M MES pH 6 and 0.15 M NaCl. The chips were subsequently immobilized with the recombinant human Hsp90 protein at a concentration of 0.5 mg/ml in 20 mM sodium acetate, pH 4.5 and then blocked with 1 M ethanolamine, pH 8.0. Araloside C was prepared as a 10 mM solution in running buffer before the experiment and diluted two‐fold by running buffer into 80, 40, 20, 10 and 0 μM before injection. The optical interference pattern was recorded as a change in optical path difference in units of nm. Data were analysed with SKi Report software.

### Cell culture and Hypoxia/Reoxygenation

The H9c2 cardiomyocyte line was obtained from the Chinese Academy of Sciences Cell Bank (Shanghai, China) and cultured as previously described 24. Briefly, H9c2 cells were cultured in high‐glucose DMEM supplemented with 10% (v/v) foetal bovine serum, 1% penicillin/streptomycin (v/v) and 2 mM l‐glutamine. The cells were maintained at 37°C with 100% relative humidity in a CO_2_ incubator containing 5% CO_2_ at 37°C.

The hypoxia/reoxygenation (H/R) procedures were modified from a previous study 19, 25. For all experiments, cells were plated at an appropriate density according to the experimental design and were grown for 24 hrs to reach 70–80% confluence before experimentation.

### Experimental protocols in H9c2 cardiomyocytes

The cultured H9c2 cardiomyocytes were randomly divided into different groups. In the control group, the H9c2 cardiomyocytes were incubated under normoxic conditions for equivalent durations with high‐glucose DMEM. The H/R group was conducted as described in the preceding section. In the Araloside C‐treated group (H/R + A‐C), the H9c2 cardiomyocytes were pre‐treated with Araloside C for 12 hrs before subjected to H/R. Inhibitor‐treated groups were processed the same as the H/R + A‐C group, but the cells were incubated with 2 μM 17‐AAG for 1 hr before they were treated with Araloside C.

### Cell viability analysis

Cell viability was determined by the 3‐(4, 5‐dimethylthiazol‐2‐yl)‐2, 5‐diphenyl tetrazolium (MTT) assay as previously described 19. Briefly, H9c2 cells were plated on 96‐well plates at a density of 1 × 104 cells/well. After designated treatment, 20 μl MTT (5 mg/ml) was added to each well and incubated for 4 hrs. The medium was then removed, and the formazan crystals were dissolved with dimethyl sulphoxide (DMSO). Absorbance was read at 570 nm on a microplate reader (TECAN Infinite M1000, Austria).

### Measurement of ROS production in cardiomyocytes

The cellular ROS levels were determined according to the manufacturer's protocol using total ROS detection kits (Enzo Life Sciences Inc., Farmingdale, NY, USA) as previously described 26. Briefly, following treatment, the cells were incubated with 100 μl of 5‐(and‐6)‐carboxy‐2′, 7′‐dichlorodihydrofluorescein diacetate (carboxy‐H2DCFDA) (25 μM final concentration) in darkness at 37°C for 30 min. Cellular DCF fluorescence intensity was determined through microplate reader with excitation wavelength of 495 nm and emission wavelength of 529 nm. The ROS level was expressed as a percentage of the control.

### Western blot

Cell lysate preparation and Western blot analysis were performed as previously described 19. The primary antibodies were against Hsp90 and β‐actin (Abcam, Cambridge, UK). Specific bands were visualized after incubation with horseradish peroxidase‐conjugated secondary antibodies by enhanced chemiluminescence using a Bio‐Rad imaging system (Bio‐Rad, Hercules, CA, USA).

### Statistical analyses

The results are expressed as the means ± standard deviation. Comparisons between >2 groups or multiple groups over time were performed using simple or repeated‐measures anova (Prism 5.00 software, GraphPad Software Inc, La Jolla, CA, USA) as appropriate. A Newman–Keuls *post hoc* test was used, except in the analysis of Langendorff data, where Bonferroni's analysis was used. Statistical significance was set at *P* < 0.05. All data are the result of at least three independent experiments.

## Results

### Araloside C improved cardiac function of I/R hearts

**Figure 2:**
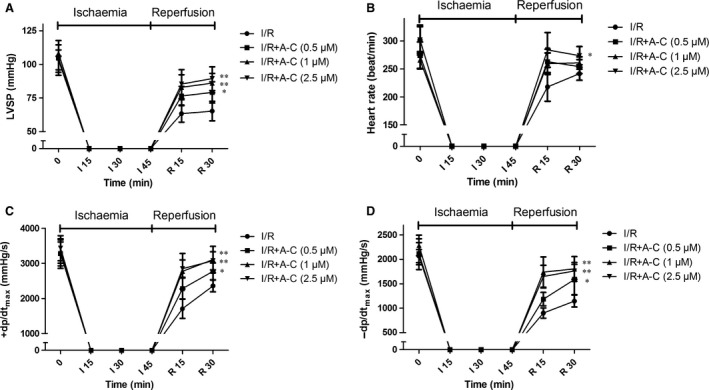
Effects of different concentrations of Araloside C on cardiac function in isolated rat hearts subjected to 45 min. of no‐flow global ischaemia followed by 30 min. of reperfusion. Hearts were exposed to Araloside C (A‐C) at concentrations of 0.5–2.5 μM for 15 min. prior to ischaemia. (**A**–**D**) Time course of cardiac functional indexes in isolated hearts during I/R. (**A**) Left ventricular developed pressure (LVDP); (**B**) heart rate; (**C**) maximum rate of LV pressure development (+d*P*/d*t*_max_); (**D**) maximum rate of LV pressure decline (–d*P*/d*t*_max_). *n* > 10 in each group, *P < 0.05 *versus* I/R; **P < 0.01 *versus* I/R.

To characterize the cardioprotective effects of Araloside C against I/R injury, we perfused isolated rat hearts with Araloside C at concentrations from 0.5 to 2.5 μM for 15 min. prior to ischaemia. Compared with the I/R group, Araloside C (0.5–2.5 μM) concentration‐dependently improved the functional recovery of the I/R hearts, including increase in the recovery of LVDP, ±d*P*/d*t*_max_ and heart rate throughout the reperfusion period, although the baseline mechanical parameters with Araloside C were not significantly different compared with those of the control condition (Fig. 2A–D). In the additional groups, we found that Araloside C treatment alone did not have a significant effect on the mechanical parameters after 90 min. of perfusion compared with those of the control group (data not shown).

### Araloside C reduced I/R‐induced myocardial oxidative stress

**Figure 3:**
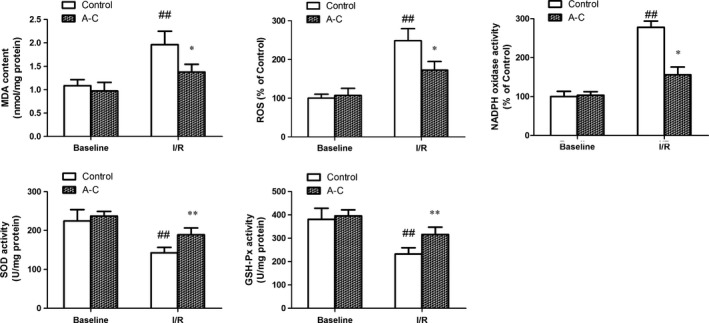
Effects of Araloside C on oxidative damage in I/R‐injured hearts. SOD: superoxide dismutase; GSH‐PX: glutathione peroxidase. MDA: malondialdehyde; ROS: reactive oxygen species; NADPH oxidase: nicotinamide adenine dinucleotide phosphate oxidase; A‐C: Araloside C. *n* > 10 in each group, ^##^ *P* < 0.01 *versus* baseline control; *P < 0.05 *versus* I/R control; **P < 0.01 *versus* I/R control.

Malondialdehyde levels were used as surrogate assays for membrane lipid oxidation, which is one of the primary events in oxidative damage 27. We found that the MDA levels, ROS levels and NADPH oxidase were significantly lower in the Araloside C‐treated (2.5 μM) group compared with those of the I/R group. Consistently, the activities of the major cellular antioxidants GSH‐Px and SOD in the rat myocardium were decreased in the I/R group and significantly elevated by treatment with Araloside C (Fig. 3).

### Araloside C improved the impairment of cardiomyocyte contractile capacity and intracellular Ca^2+^ homoeostasis induced by I/R

**Figure 4:**
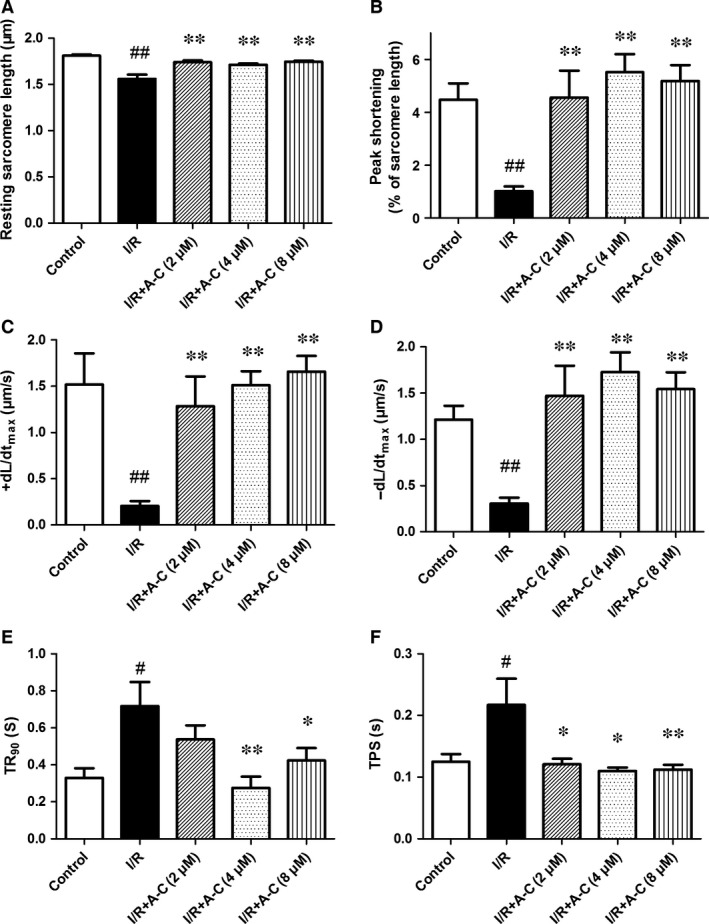
Effect of Araloside C on sarcomere contraction of adult rat cardiomyocytes during I/R. (**A**) Resting sarcomere length; (**B**) peak shortening (% of resting cell length); (**C**) maximal velocity of shortening (+d*L*/d*t*); (**D**) maximal velocity of re‐lengthening (−d*L*/d*t*); (**E**) time‐to‐90% re‐lengthening (TR90); (**F**) time‐to‐peak shortening (TPS). A‐C: Araloside C. All data are expressed as the means ± S.D., *n* = 28 to 35 cells from three rats per group, ^#^ *P* < 0.05 *versus* control; ^##^ *P* < 0.01 *versus* control; *P < 0.05 *versus* I/R; **P < 0.01 *versus* I/R.

Our further assessment of the cardiomyocyte mechanics revealed that Araloside C could improve post‐ischaemic cell shortening and Ca^2+^ transients from 2 to 8 μM. I/R markedly depressed the peak shortening (PS) amplitude and the maximal velocity of shortening/re‐lengthening (± d*L*/d*t*), accompanied by a prolonged duration of re‐lengthening (TR_90_) and duration of shortening (TPS). However, all of these effects were significantly attenuated by pre‐treatment with Araloside C (Fig. 4).

**Figure 5:**
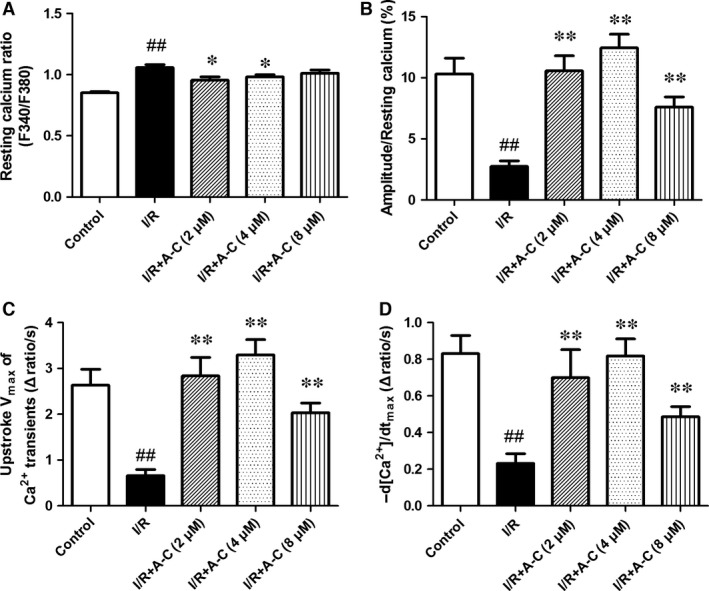
Effect of Araloside C on Ca^2+^ transient of adult rat cardiomyocytes during I/R. (**A**) Resting intracellular Ca^2+^ levels; (**B**) amplitude of Ca^2+^ transients; (**C**) maximum decay velocity of Ca^2+^ transients; (**D**) Ca^2+^ transient decay rate. A‐C: Araloside C. F340/F380, fluorescence ratio of 340 nm to 380 nm. All data are expressed as the means ± S.D., *n* = 28–35 cells from three rats per group, ^##^ *P* < 0.01 *versus* control; *P < 0.05 *versus* I/R; **P < 0.01 *versus* I/R.

I/R induced a significant increase in resting levels of Ca^2+^ transients that represent the diastolic cytosolic Ca^2+^ content, but this increase was markedly restored by Araloside C (Fig. 5A). The amplitude of the Ca^2+^ transients and the maximum upstroke velocity (*V*_max_) of Ca^2+^ transients were decreased after I/R, while Araloside C significantly attenuated those suppressions (Fig. 5B and C). The decay of [Ca^2+^]_i_, representing the speed of Ca^2+^ removal from the cytoplasm, mainly via sarco/endoplasmic reticulum Ca^2+^‐ATPase (SERCA), was significantly prolonged during I/R. Araloside C reduced the decay of [Ca^2+^]_i_ close to the control level, suggesting an increase in SERCA activity (Fig. 5D).

### Target prediction

The molecular targets of Araloside C were predicted using the web‐based DDI‐CPI tool and Discovery Studio software, respectively. More than seventy potential targets were identified and annotated to have significant relationships with the pharmacologic effects of Araloside C (Table 1). Among these targets, we focused on the heat shock protein 90 (Hsp90), which is a critical target in the protection of the myocardium from I/R injury.

**Table 1:** Results of Araloside C‐Hsp90 interactome by DDI‐CPI and Discovery Studio

DDI‐CPI			Discovery Studio		
PDB ID	Putative target	Docking Score	PDB ID	Putative target	FitValue
3EKO	Heat shock protein HSP 90‐alpha	−8.8	1 amw	ADP‐binding site in the Hsp90 molecular chaperone	0.824059
3NMQ	Heat shock protein HSP 90‐beta	−8.3			

### Docking analysis of the interaction between Araloside C and Hsp90

**Figure 6:**
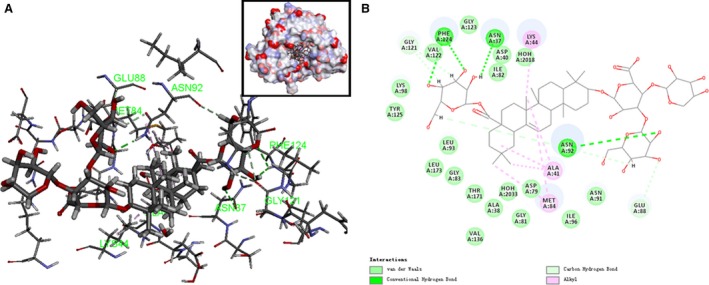
Modelling study of the structure of Araloside C binding to Hsp90 protein. (**A**) Three‐dimensional modelling of Araloside C binding within the ATP/ADP‐binding domain of Hsp90. (**B**) Two‐dimensional ligand interaction diagram of Araloside C and Hsp90.

Following prediction of the molecular target, we examined the possible interaction between Araloside C and the Hsp90 protein using Discovery Studio 4.5. Molecular modelling of Araloside C revealed that it was bound deeply into the binding cavity of Hsp90 and showed important conventional hydrogen bond interaction with the amino acid residues Asn 37, Asn92 and Phe124 (Fig. 6A). As seen in Figure 6B, Gly 121 and Glu 88 are responsible for binding the glycosyl moiety in the extracted Araloside C with carbon–hydrogen bond interactions. Additionally, it seemed that the presence of triterpenoid aglycone backbone on Araloside C led to the formation of weak alkyl interaction with amino acid residues Lys 44, Ala 41 and Met 84, which are important to the binding site. The LibDockScore (133.521) was obtained based on the virtual calculation of the interaction of Araloside C with the targeted Hsp90 protein.

### Araloside C binds to Hsp90 in a dose‐dependent manner

**Figure 7:**
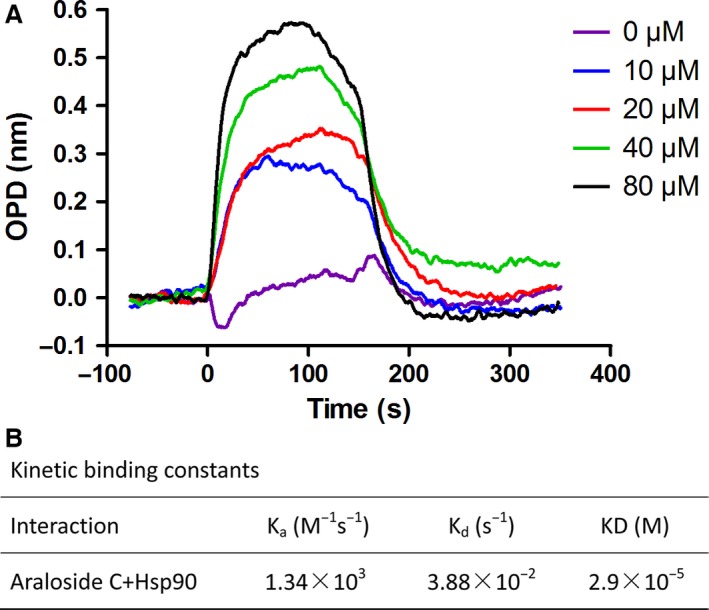
The binding kinetic of Araloside C to Hsp90 tested by Nanopore optical interferometry. Hsp90 immobilized to a carboxy chip was provided with the Araloside C at concentrations varying from 0 μM to 80 μM in a two‐fold dilution series. (**A**) Representative binding curves for Araloside C binding to Hsp90. (**B**) Summary of calculated kinetic binding constants for the interaction between Araloside C and Hsp90.

To further characterize the interaction between Araloside C and Hsp90, we determined the binding affinity kinetic constants using SKi Pro nanopore optical interferometry. Hsp90 was immobilized to the nanoporous silicon chip, and the Araloside C was provided at varying concentrations. As shown in Figure 7, Araloside C bound to Hsp90 in a dose‐dependent manner. The resultant KD constant of Araloside C binding to Hsp90 was 29 μM, indicating the likelihood of direct binding of Araloside C to Hsp90.

### Hsp90 contributes to the cytoprotection of Araloside C against H/R injury in H9c2 cardiomyocytes

**Figure 8:**
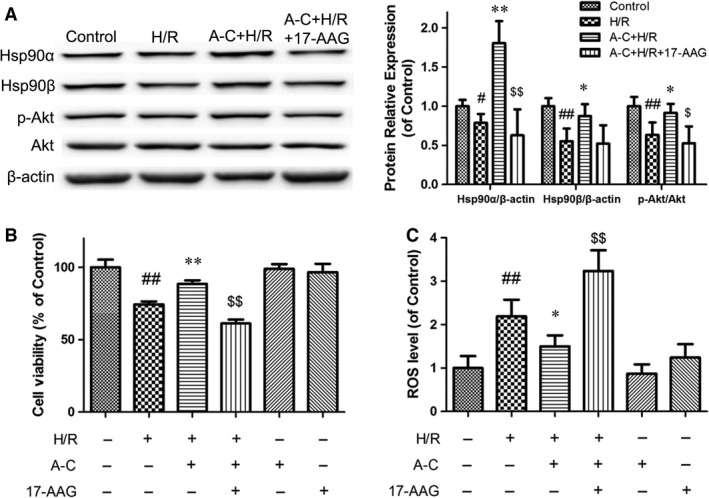
Effects of Hsp90 on the protection of Araloside C against H/R‐induced cell injury. (**A**) Effects of Araloside C on Hsp90 expression and Akt phosphorylation levels in H/R‐treated cardiomyocytes. Cell lysates were harvested, and Western blot analysis was performed. β‐actin expression was examined as the protein loading control. (**B**) Effects of Araloside C and the Hsp90 inhibitor 17‐AAG on cell viability in H/R‐treated cardiomyocytes. Cardiomyocyte viability was assessed using the MTT assay. (**C**) Effects of Araloside C and 17‐AAG on reactive oxygen species (ROS) levels in H/R‐treated cardiomyocytes. The intracellular ROS levels were measured with a fluorometric assay. H/R, Hypoxia/reoxygenation; A‐C, Araloside C. The data are expressed as means ± S.D. from three independent experiments. ^#^ *P* < 0.05 *versus* control; ^##^ *P* < 0.01 *versus* control; *P < 0.05 *versus* H/R‐treated cells; **P < 0.01 *versus* H/R‐treated cells; $P < 0.05 *versus* H/R + A‐C‐treated cells; $$*P* < 0.01 *versus* H/R + A‐C‐treated cells.

Studies have shown that overexpression of HSP90 can protect cardiac myocytes against ischaemia/reperfusion injury 8. In Araloside C‐treated H9c2 cells, Hsp90 expression was increased in a time‐dependent manner (Fig. S2). To further investigate the potential role of Hsp90 in Araloside C‐induced cardioprotection, we then detected the effects of Araloside C on Hsp90 expression levels in H/R‐treated cardiomyocytes. Figure 8A shows that Araloside C treatment significantly inhibited the down‐regulation of the H/R‐induced Hsp90 (both Hsp90α and Hsp90β) expression. To determine whether such elevated expression of Hsp90 can contribute to Araloside C‐mediated protection against H/R‐induced cytotoxicity, a selective inhibitor of Hsp90, 17‐AAG, was utilized. Araloside C considerably attenuated H/R‐induced cytotoxicity, evidenced by enhanced phosphorylation of pro‐survival protein Akt, improved cell viability and decreased ROS level. However, this cytoprotection of Araloside C treatment was reduced by 17‐AAG pre‐treatment (Fig. 8B and C), which suggested that Hsp90 is involved in the protective effect of Araloside C against H/R injury in H9c2 cells.

## Discussion

In the present study, we not only determined the concentration‐dependent cardioprotection of Araloside C, but also identified the possible molecular target of Araloside C. We observed the following: (*i*) Araloside C ameliorates myocardial dysfunction and depresses oxidative stress in I/R hearts; (*ii*) Araloside C improves cardiomyocyte contraction and Ca^2+^ transients during I/R; and (*iii*) the cardioprotective effect of Araloside C is dependent on its binding to the Hsp90 protein. These findings provide novel evidence and insights related to the role of Araloside C against myocardial I/R injury.

Numerous reports indicate that myocardial ischaemia/reperfusion would cause a sharp decline in cardiac function, leading to a marked decrease in LVSP, heart rate and ventricular contractility 20, 28. The present study has provided evidence that Araloside C pre‐treatment concentration‐dependently promotes the recovery of I/R‐induced myocardial dysfunction in rats. More specifically, at the end (30 min.) of reperfusion, LVSP was 86.11 + 8.82% of baseline levels in the Araloside C + I/R treatment group, while Elatoside C, another major triterpenoid saponin isolated from *A. elata*, was 75.66 + 10.2% of baseline levels at the 30 min. of reperfusion in our previous report 20. It seems that the effect of Araloside C is a little stronger than that of Elatoside C on cardiac function of the I/R hearts. However, there is no statistically significant difference between Araloside C pre‐treatment group and Elatoside C group. After initial investigation of the effect of Araloside C on the whole heart, we then further observed the effect on the cellular level. We demonstrated that Araloside C significantly improves contractile function in I/R cardiomyocytes. Our observations indicate that Araloside C has a beneficial effect on cardiac performance during I/R.

Excessive ROS production and calcium overload are the two proposed mechanisms of cardiac dysfunction in IR injury 2, 29. ROS overproduction causes damage to the cell membranes in rat hearts, subsequently causes cell membrane lipid peroxidation and results in myocardial structural failure 30. In this study, we investigated the effects of Araloside C on oxidative stress in I/R hearts. Our results clearly showed that SOD activity and GSH‐Px were significantly elevated as a result of Araloside C pre‐treatment, whereas these effects were accompanied by significantly decreased levels of MDA and ROS, indicating that the cardioprotective effects of Araloside C may possibly be ascribed to its direct antioxidant property. Alternatively, reperfusion can lead to a dramatic rise of [Ca^2+^]_i_ due to abnormal Ca^2+^ handling 29, 31. The present data show that Araloside C significantly improves cell contraction and Ca^2+^ transients in isolated cardiomyocytes during I/R. The reduced activity of ryanodine receptors (RyRs) at the end of reperfusion would be detrimental to the contractility due to the depression of Ca^2+^ release from the sarcoplasmic reticulum(SR) 32. Here, we show that the amplitude of Ca^2+^ transients and the speed of Ca^2+^ release via RyRs were significantly faster in the Araloside C group, suggesting that Araloside C improved the activity of RyRs. The decay of [Ca^2+^]_i_ represents the speed of Ca^2+^ uptake mainly via SERCA 33. The depressed decay rate of [Ca^2+^]_i_ transience stimulated by Araloside C can be interpreted at least in part to the preserved protein content and activity of the SERCA during I/R. Thus, we speculate that Araloside C may suppress I/R‐induced Ca^2+^ overload by reserving the protein content and function of RyRs and SERCA, thereby maintaining relatively normal Ca^2+^ homoeostasis during I/R, leading to improvement in contractility. Taken together, the beneficial effects of Araloside C on myocardial I/R injury seem to be largely due to the attenuation of oxidative stress and the maintenance of [Ca^2+^]_i_ homoeostasis.

Bioinformatic approaches have become a powerful tool for interpretation and prediction of the potential biological activities of drugs and their targets 34, 35. We therefore used a DDI‐CPI tool and Discovery Studio software to predict the potential targets of Araloside C. Our findings show that Araloside C can modulate a number of functional protein targets. Among the number of targets identified by screening, Hsp90, one of the most abundant and conserved molecular chaperones, has been shown to protect the myocardium against I/R injury 8. Hsp90 is essential for the integrity and function of numerous signal transduction molecules and is identified directly as the effector allowing activation of the pro‐survival PI3K/Akt pathway 36, 37. Previous studies have shown that Hsp90 plays a vital role in ischaemic pre‐conditioning and post‐conditioning 7, 38. Targeted overexpression of Hsp90 in myocardium reduces infarct size and myocardial dysfunction 9. However, the Hsp90 inhibitor increases cardiomyocyte apoptosis through reducing pro‐survival Akt signalling and enhancing the activity of pro‐apoptotic BAD 39. In isolated hearts, Hsp90 also has been shown to facilitate protein kinase Cε targeting to mitochondria, ultimately attenuating I/R‐induced cardiomyocyte apoptosis and necrosis 40. Notably, the recent study shows that Hsp90 appears to modulate the inhibitory function of HAX‐1 on calcium cycling and supports certain of its cardioprotective effects at the ER/SR 41. Moreover, the HAX‐1/Hsp90 complex may also inhibit activation of the mPTP via regulation of cyclophilin‐D (Cyp‐D) levels and promote cardiomyocyte survival upon oxidative stress and calcium overload in ischaemia/reperfusion injury 42.

Because Hsp90 played a crucial role to modulate cardioprotection against myocardial I/R injury and was predicted to be a potential target of Araloside C, we selected it for our subsequent confirmative assays. Molecular docking is a commonly and frequently applied technique to predict the specific positioning of a ligand in the active site of a protein 43, 44. The molecular docking study using the Discovery Studio program showed that Araloside C was indeed docked closely in the active site of Hsp90. Moreover, we have characterized the kinetic association constants between Araloside C and Hsp90 using nanopore optical interferometry. Our results demonstrated that Araloside C was able to bind to the Hsp90 protein *in vitro* in a dose‐dependent manner with the KD 29 μM. Because our studies indicated an interaction between Araloside C and Hsp90, we also investigated the effects of Hsp90 on the protection of Araloside C against hypoxia/reoxygenation (H/R) injury for H9c2 cardiomyocytes. These experiments revealed that treatment with Araloside C significantly attenuated the cytotoxicity and the down‐regulation of Hsp90 expression induced by H/R, which was consistent with the findings in IR‐injured isolated hearts (Fig. S3). Notably, inhibition of Hsp90 via the Hsp90 inhibitor 17‐AAG significantly blocked the Araloside C‐induced cardioprotective effect against H/R injury, as demonstrated by inhibited Akt activation, decreased cell viability and increased ROS levels. Cumulatively, the computational and experimental data presented here highly support the speculation that Araloside C may help attenuate myocardial I/R injury by interacting with Hsp90. Nevertheless, the detail of the mechanism for Araloside C interaction with Hsp90 needs to be further examined and functionally validated in the future.

In conclusion, the present study demonstrated that Araloside C provides cardioprotection following I/R‐induced myocardial injury by reducing ROS generation and Ca^2+^ overload. It is highly likely that such cardioprotection is due to the ability of Araloside C to bind to the Hsp90 protein, specifically interacting with the ATP/ADP‐binding domain of Hsp90. These observations provide evidence supporting Araloside C as a promising therapeutic candidate for myocardial I/R injury in cardiac surgery and ischaemic heart disease.

## Conflicts of interest

The authors confirm that there are no conflicts of interest.

## Supporting information


**Appendix S1** Methods.
**Figure S1** HPLC (UV) chromatogram of Araloside C.
**Figure S2** Effects of Araloside C on Hsp90 expression levels in H9c2 cardiomyocytes.
**Figure S3** Effects of Araloside C on Hsp90 expression levels in IR‐injured isolated rat hearts.
