# Supplementary material for: Protective effects of Araloside C against myocardial ischaemia/reperfusion injury: potential involvement of heat shock protein 90
Source: J Cell Mol Med. 2017 Feb 22;21(9):1870–80. doi: 10.1111/jcmm.13107 (PMC5571541; doi:10.1111/jcmm.13107)

**SUPPLEMENTARY MATERIAL**

**Supplementary Methods**

**Preparation of Araloside C**

Araloside C was separated from *Aralia elata* using various column chromatographies particularly the semi-preparative HPLC method in our lab [[1](#_ENREF_1)]. The chemical structure of Araloside C was established by analyzing its NMR data and comparing with reported literature. Its purity (>96%) was determined by HPLC equipped with a UV detector with an Aglient Eclipse XDB-C18 column (5µm, 4.6 mm i.d. × 250 mm) (Supplementary Fig. 1**)**.

**Supplemental References**

[1] Zhang JX, Tian Y, Zhong XM, Sun GB, Sun XB, XD. X. Study on Saponins Chemical Components from Aralia elata. China Pharmacy. 2013;24:1380-2.

**Supplemental Figure Legends**

**Supplementary Fig. 1** HPLC (UV) chromatogram of Araloside C


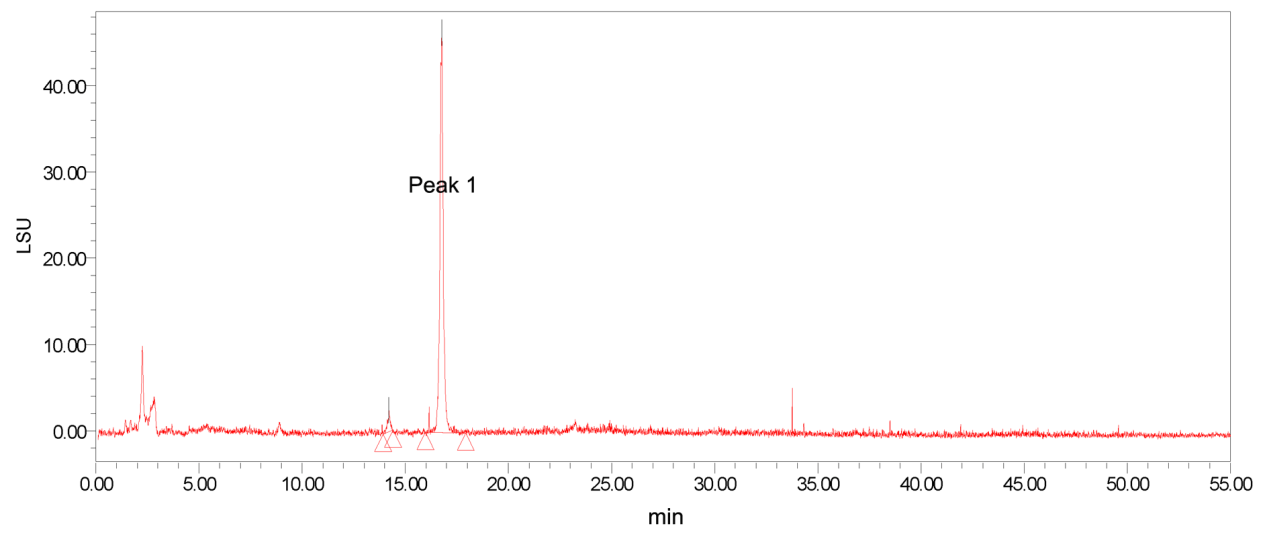


| Peak | t_R_(min) | Area | Height | %Area |
| --- | --- | --- | --- | --- |
| 1 | 16.764 | 540664 | 46424 | 96.12 |

**Supplementary Fig. 2** Effects of Araloside C on Hsp90 expression levels in H9c2 cardiomyocytes. (A) Hsp90α/β-actin; (B) Hsp90β/β-actin. H9c2 cells were treated with Araloside C (12.5 μM) for various times as indicated. β-actin expression was examined as the protein loading control. A-C, Araloside C. The data are expressed as means ± SD from three independent experiments. #p< 0.05 versus control; ##p < 0.01 versus control.

**
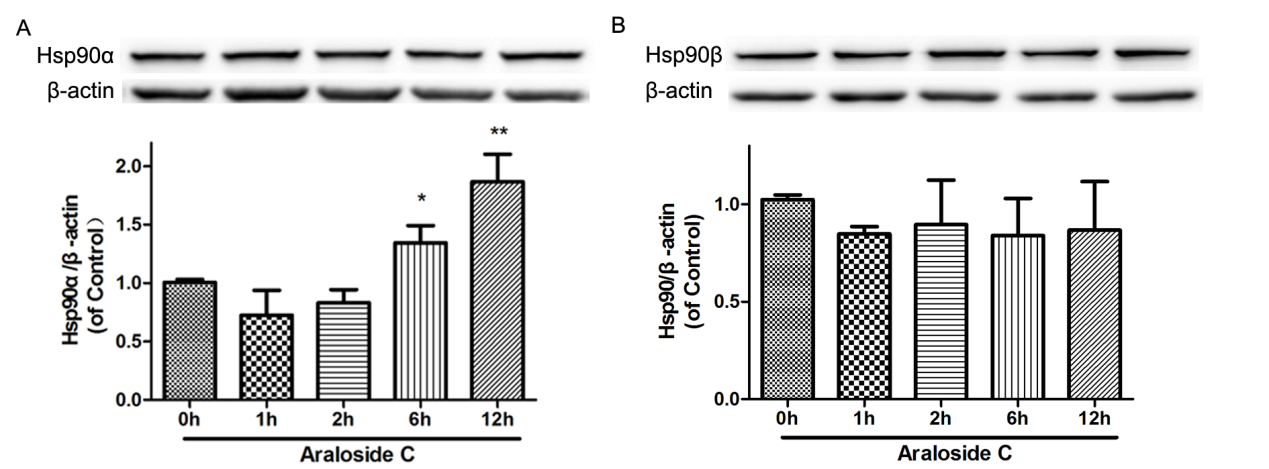
**

**Supplementary Fig. 3** Effects of Araloside C on Hsp90 expression levels in IR-injured isolated rat hearts. β-actin expression was examined as the protein loading control. A-C, Araloside C. #p< 0.05 versus control; ##p < 0.01 versus control; *p < 0.05 versus IR group; **p < 0.01 versus IR group.


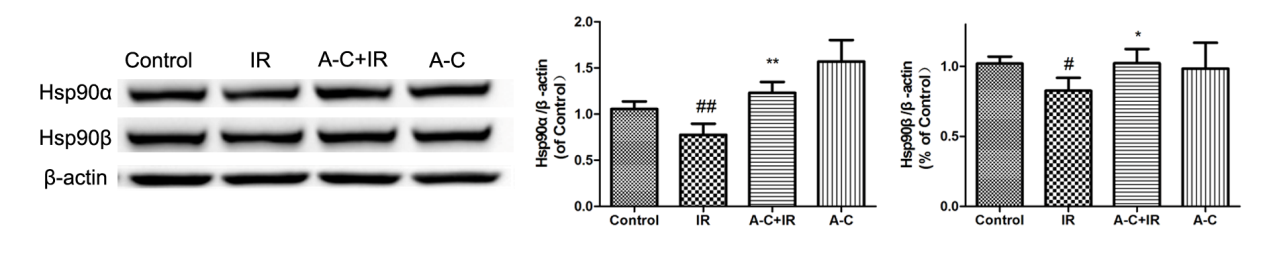

Supplement: Supplementary file 1 — Appendix S1 Methods. Figure S1 HPLC (UV) chromatogram of Araloside C. Figure S2 Effects of Araloside C on Hsp90 expression levels in H9c2 cardiomyocytes. Figure S3 Effects of Araloside C on Hsp90 expression levels in IR‐injured isolated rat hearts. [file JCMM-21-1870-s001.docx]
